# Supplementary material for: Modeling glioblastoma heterogeneity as a dynamic network of cell states
Source: Mol Syst Biol. 2021 Sep 16;17(9):e10105. doi: 10.15252/msb.202010105 (PMC8444284; doi:10.15252/msb.202010105)
Supplement: Supplementary file 6 — Source Data for Figure 5 [file MSB-17-e10105-s004.zip › Figure5A_sourcedata/GSEA_3071/nosigdb_stateI.GseaPreranked.1622795145664/GANDAL_AUTISM_UP_PMID29439242.html]

Details for gene set GANDAL\_AUTISM\_UP\_PMID29439242[GSEA]

|  || Dataset | state13071 |
| Phenotype | NoPhenotypeAvailable |
| Upregulated in class | na\_pos |
| GeneSet | GANDAL\_AUTISM\_UP\_PMID29439242 |
| Enrichment Score (ES) | 0.5532111 |
| Normalized Enrichment Score (NES) | 2.8391552 |
| Nominal p-value | 0.0 |
| FDR q-value | 0.0 |
| FWER p-Value | 0.0 |
Table: GSEA Results Summary

  

Fig 1: Enrichment plot: GANDAL\_AUTISM\_UP\_PMID29439242      
 Profile of the Running ES Score & Positions of GeneSet Members on the Rank Ordered List

  

| PROBE | GENE SYMBOL | GENE\_TITLE | RANK IN GENE LIST | RANK METRIC SCORE | RUNNING ES | CORE ENRICHMENT || 1 | SPP1 |  |  | 0 | 2.808 | 0.0661 | Yes |
| 2 | MGP |  |  | 1 | 2.652 | 0.1285 | Yes |
| 3 | TGFBI |  |  | 17 | 1.674 | 0.1635 | Yes |
| 4 | RARRES3 |  |  | 22 | 1.575 | 0.1994 | Yes |
| 5 | COL4A1 |  |  | 28 | 1.524 | 0.2338 | Yes |
| 6 | TIMP1 |  |  | 47 | 1.381 | 0.2611 | Yes |
| 7 | ZFP36L1 |  |  | 97 | 1.096 | 0.2727 | Yes |
| 8 | COL8A1 |  |  | 111 | 1.056 | 0.2938 | Yes |
| 9 | NQO1 |  |  | 113 | 1.045 | 0.3181 | Yes |
| 10 | EMP1 |  |  | 122 | 1.012 | 0.3396 | Yes |
| 11 | FAM129A |  |  | 135 | 0.953 | 0.3585 | Yes |
| 12 | SERPINH1 |  |  | 157 | 0.902 | 0.3736 | Yes |
| 13 | IGFBP5 |  |  | 198 | 0.833 | 0.3817 | Yes |
| 14 | AEBP1 |  |  | 236 | 0.761 | 0.3889 | Yes |
| 15 | GBP1 |  |  | 248 | 0.736 | 0.4030 | Yes |
| 16 | TM4SF1 |  |  | 249 | 0.735 | 0.4203 | Yes |
| 17 | EMP3 |  |  | 260 | 0.726 | 0.4345 | Yes |
| 18 | IFITM3 |  |  | 268 | 0.714 | 0.4493 | Yes |
| 19 | S100A11 |  |  | 275 | 0.707 | 0.4641 | Yes |
| 20 | P4HA1 |  |  | 291 | 0.688 | 0.4760 | Yes |
| 21 | MYOF |  |  | 310 | 0.663 | 0.4864 | Yes |
| 22 | DSE |  |  | 312 | 0.659 | 0.5016 | Yes |
| 23 | ACKR3 |  |  | 332 | 0.641 | 0.5112 | Yes |
| 24 | PALLD |  |  | 342 | 0.636 | 0.5235 | Yes |
| 25 | DDIT4L |  |  | 344 | 0.633 | 0.5381 | Yes |
| 26 | LY96 |  |  | 451 | 0.547 | 0.5204 | Yes |
| 27 | HSPA1A |  |  | 566 | 0.473 | 0.4985 | Yes |
| 28 | SLC16A3 |  |  | 570 | 0.470 | 0.5087 | Yes |
| 29 | DTNA |  |  | 587 | 0.460 | 0.5149 | Yes |
| 30 | MTHFD2 |  |  | 588 | 0.459 | 0.5257 | Yes |
| 31 | VCAM1 |  |  | 603 | 0.450 | 0.5322 | Yes |
| 32 | HMOX1 |  |  | 623 | 0.443 | 0.5371 | Yes |
| 33 | YAP1 |  |  | 657 | 0.430 | 0.5377 | Yes |
| 34 | S100A10 |  |  | 673 | 0.422 | 0.5433 | Yes |
| 35 | GBP2 |  |  | 674 | 0.422 | 0.5532 | Yes |
| 36 | IFITM2 |  |  | 836 | 0.370 | 0.5153 | No |
| 37 | DDIT4 |  |  | 865 | 0.361 | 0.5157 | No |
| 38 | ITGA5 |  |  | 874 | 0.357 | 0.5218 | No |
| 39 | SERPINB1 |  |  | 918 | 0.346 | 0.5175 | No |
| 40 | IFI6 |  |  | 923 | 0.344 | 0.5245 | No |
| 41 | TNFRSF10B |  |  | 989 | 0.329 | 0.5134 | No |
| 42 | IFI16 |  |  | 1062 | 0.315 | 0.5000 | No |
| 43 | GPNMB |  |  | 1070 | 0.312 | 0.5053 | No |
| 44 | CEBPD |  |  | 1310 | 0.267 | 0.4424 | No |
| 45 | CYR61 |  |  | 1318 | 0.265 | 0.4466 | No |
| 46 | MSN |  |  | 1379 | 0.255 | 0.4353 | No |
| 47 | CD44 |  |  | 1430 | -0.251 | 0.4267 | No |
| 48 | TGM2 |  |  | 1444 | -0.252 | 0.4289 | No |
| 49 | CDKN1A |  |  | 2083 | -0.332 | 0.2521 | No |
| 50 | PNP |  |  | 2223 | -0.358 | 0.2203 | No |
| 51 | UCP2 |  |  | 2498 | -0.413 | 0.1507 | No |
| 52 | PDLIM4 |  |  | 2861 | -0.525 | 0.0583 | No |
| 53 | STC1 |  |  | 3349 | -1.043 | -0.0581 | No |
| 54 | ADAMTS9 |  |  | 3407 | -1.260 | -0.0449 | No |
| 55 | HIST1H1C |  |  | 3427 | -1.335 | -0.0190 | No |
| 56 | CRYAB |  |  | 3475 | -1.829 | 0.0104 | No |
Table: GSEA details [plain text format]

  

Fig 2: GANDAL\_AUTISM\_UP\_PMID29439242: Random ES distribution      
 Gene set null distribution of ES for **GANDAL\_AUTISM\_UP\_PMID29439242**
